# Non-caloric sweetener provides magnetic resonance imaging contrast for cancer detection

**DOI:** 10.1186/s12967-017-1221-9

**Published:** 2017-05-30

**Authors:** Puneet Bagga, Mohammad Haris, Kevin D’Aquilla, Neil E. Wilson, Francesco M. Marincola, Mitchell D. Schnall, Hari Hariharan, Ravinder Reddy

**Affiliations:** 1 Department of Radiology, Center for Magnetic Resonance and Optical Imaging, Perelman School of Medicine, University of Pennsylvania, 422 Curie Blvd, B1-Stellar-Chance Laboratories, Philadelphia, PA USA; 2 Research Branch, Sidra Medical and Research Center, Doha, Qatar

**Keywords:** CEST, MRI, Glioma, Cancer, Gadolinium, Sucralose

## Abstract

**Background:**

Image contrast enhanced by exogenous contrast agents plays a crucial role in the early detection, characterization, and determination of the precise location of cancers. Here, we investigate the feasibility of using a non-nutritive sweetener, sucralose (commercial name, Splenda), as magnetic resonance imaging (MRI) contrast agent for cancer studies.

**Methods:**

High-resolution nuclear-magnetic-resonance spectroscopy and MR studies on sucralose solution phantom were performed to detect the chemical exchange saturation transfer (CEST) property of sucralose hydroxyl protons with bulk water (sucCEST). For the animal experiments, female Fisher rats (F344/NCR) were used to generate 9L-gliosarcoma model. MRI with CEST experiments were performed on anesthetized rats at 9.4 T MR scanner. Following the baseline CEST scans, sucralose solution was intravenously administered in control and tumor bearing rats. CEST acquisitions were continued during and following the administration of sucralose. Following the sucCEST, Gadolinium-diethylenetriamine pentaacetic acid was injected to perform Gd-enhanced imaging for visualizing the tumor.

**Results:**

The sucCEST contrast in vitro was found to correlate positively with the sucralose concentration and negatively with the pH, indicating the potential of this technique in cancer imaging. In a control animal, the CEST contrast from the brain was found to be unaffected following the administration of sucralose, demonstrating its blood–brain barrier impermeability. In a 9L glioma model, enhanced localized sucCEST contrast in the tumor region was detected while the unaffected brain region showed unaltered CEST effect implying the specificity of sucralose toward the tumorous tissue. The CEST asymmetry plots acquired from the tumor region before and after the sucralose infusion showed elevation of asymmetry at 1 ppm, pointing towards the role of sucralose in increased contrast.

**Conclusions:**

We show the feasibility of using sucralose and sucCEST in study of preclinical models of cancer. This study paves the way for the potential development of sucralose and other sucrose derivatives as contrast agents for clinical MRI applications.

**Electronic supplementary material:**

The online version of this article (doi:10.1186/s12967-017-1221-9) contains supplementary material, which is available to authorized users.

## Background

Medical imaging can provide morphological, structural, metabolic and functional information of tumors and is an essential part of cancer clinical protocols. In order to accurately detect and characterize tumors, exogenous contrast agents are often used. Positron emission tomography (PET) imaging of ^18^fluoro-2-deoxy-glucose (^18^FDG), an analogue of glucose, is most widely used to characterize cancers based on the high uptake rate and glycolytic activity of tumors compared to healthy tissue [1–5]. Although ^18^FDG-PET provides valuable functional and metabolic information of cancers, it may be limited by the lack of specificity of ^18^FDG uptake and the patients exposure to ionizing radiation [6].

Magnetic resonance imaging (MRI) coupled with administration of gadolinium based contrast agents (GBCAs) provides exquisite contrast between normal and tumorous tissues without exposure to ionizing radiation and helps with clinical decision-making [7]. However, recent studies have reported the deposition of GBCAs in brain and bone matrix found by MRI and mass spectrometry [8–13]. Further studies are required to evaluate the long-term effects of gadolinium detected in the brain tissues or other organs on normal functioning of the organs. This provides an impetus to explore new MRI contrast agents that are non-toxic and non-metabolized. Ideal contrast agents would also be inexpensive.

Magnetic resonance imaging based on the chemical exchange saturation transfer (CEST) effect has gained widespread attention for its ability to image certain metabolites indirectly at high resolution [14–19]. In CEST, a long, frequency-selective radiofrequency pulse saturates the labile protons of a metabolite solute. The exchange of the saturated magnetization of the solute with the bulk water protons leads to a reduction in the bulk water signal compared to the signal without saturation [20–23]. The CEST method has been shown to provide higher sensitivity than direct observation with traditional proton MR spectroscopy (MRS) and was applied in monitoring the changes in metabolite and macromolecular levels in various human diseases [15, 24–28]. Recently, Glucose and its analogues have been used as CEST contrast agents (glucoCEST) to image cancers in animal models [17, 29–31] and human cancer patients [32]. However, there are a couple of confounding factors in the glucoCEST contrast because of how readily glucose is metabolized, and care must be taken with regards to differences in its metabolism in healthy and tumorous tissues, glucose’s metabolic products, and the accumulation in the extracellular and extravascular. The extent of these contributions to the glucoCEST contrast is currently unknown [33]. Nonetheless, the CEST arising in the tumor region following the glucose administration has been aptly labeled as glucoCEST.

In this study, we demonstrate the feasibility of using the popular sweetener sucralose (commercial name “Splenda”) as an MRI contrast agent to detect cancer. Sucralose does not metabolize but accumulates into tumor tissue due to the enhanced permeability and retention effect, and it exhibits CEST contrast through its labile hydroxyl protons. We termed this new method as ‘*sucCEST’*. The concentration and pH dependence of sucCEST contrast was measured in vitro in solution phantoms, and the sucCEST contrast was evaluated in a rat brain gliosarcoma model and compared with the gadolinium-diethylenetriamine pentaacetic acid (Gd-DTPA) contrast enhanced image. Finally, the application of sucCEST in cancer and other pathological conditions in humans is discussed.

## Methods

### Phantom preparations

For high-resolution ^1^H NMR spectra, 200 mM of sucralose (Sigma Aldrich, USA) solution was prepared in phosphate buffered saline (PBS) at pH 7. For imaging, phantoms were prepared in PBS and experiments were performed at 37 °C. To measure the pH dependence of sucCEST, phantoms with 10 mM sucralose concentration in PBS were prepared at a varying pH from 6.6 to 7.4 in step of 0.2 pH unit. The pH was adjusted using 1 N NaOH/HCl. For measuring concentration dependence of sucCEST contrast, phantoms with 2, 4, 6, 8, and 10 mM concentrations of sucralose were prepared in PBS at pH 7.

To obtain the SplendaCEST, we purchased Splenda from local market and prepared 0.1, 0.3 and 0.5% of Splenda solutions at pH 7.

### Phantom imaging

High-resolution ^1^H NMR phantom experiments from 200 mM sucralose solution were performed on a vertical bore Bruker Avance DMX 400 MHz spectrometer (Bruker Corporation, Germany) equipped with a 5 mm PABBI proton probe using TR = 4 s and 128 averages. The proton MRS spectrum was gathered at different temperatures (5, 15, 25, and 37 °C).

The sucCEST imaging of phantom was performed on a 9.4 T, 30 cm horizontal bore magnet (Agilent, USA) interfaced to a Varian console, with a 20-mm volume coil (M2M Imaging, USA) using a custom-programmed GRE readout pulse sequence with a frequency selective continuous wave preparation pulse for saturation. The sequence parameters were as follows: field of view (FOV) = 20 × 20 mm^2^, slice thickness = 10 mm, flip angle (FA) = 15°, repetition time (TR) = 6.2 ms, echo time (TE) = 2.9 ms, matrix size = 128 × 128. Saturation was applied every 15 s and immediately followed by 128 segment acquisitions before a long delay to allow for T1 recovery. CEST images were collected using variable saturation lengths (1 through 3 s) and saturation pulse amplitudes (B_1rms_: 2.35, 3.5, 4.7, 5.9, 7, 8.2, 9.4, 10.6, 11.7 µT). For concentration and pH dependent studies, CEST images were collected using 1 s saturation pulse at B_1rms_ of 7 µT for multiple frequencies (−3.6 to +3.6 ppm in 0.2 ppm steps) from bulk water.

B_0_ and B_1_ field maps were also gathered and used to correct the CEST contrast map using the methods described previously [16, 34]. Briefly, CEST data is acquired from −1 to 1 ppm at step size of 0.2 ppm to find the spatial dependence of the frequency of water. This spatially-dependent frequency shift is then used to correct the sucCEST z-spectra using a quadratic polynomial interpolation.

For B_1_ correction, two images were obtained using preparation square pulses with duration (τ) and prescribed flip angles of 30° and 60°. The RF pulse amplitude for a 30° flip angle was used as the reference B_1_ or B_1ref_. B_1_ maps were generated by solving the equation:

$$\frac{{{ \cos }(2\phi)}}{{{ \cos }(\phi)}} = \frac{{S(2\phi)}}{{S(\phi)}}$$

where S (*ϕ*) and S (2*ϕ*) denote pixel signals in an image with preparation flip angle *ϕ* and 2*ϕ* respectively. From the flip angle map, a B_1_ field map can be obtained using the relation, B_1_ = ϕ*(360τ)^−1^. B_1_ is then corrected assuming a linear dependence of sucCEST contrast.

### Rat tumor model preparation

To validate the sucCEST in vivo, 9L-gliosarcoma rat brain tumor model was used. It is well known that brain tumors disrupt the function of blood–brain barrier (BBB) locally in a nonhomogeneous manner [35]. The compromised BBB and enhanced permeability of tumor vasculature will enable the nonhomogeneous distribution of the injected sucralose in the tumor region.

To develop intracranial tumors, rat gliosarcoma cells (9L) were used. Syngeneic female Fisher rats (F344/NCR, 4–6 weeks old) weighing 130–150 g were used as described previously [36]. General anesthesia was induced using 2% isoflurane mixed with 1 l/min oxygen followed by 1–2% isoflurane. A 10 µl suspension of 50,000 9L cells in PBS was injected into the cortex at a depth of 3 mm with a Hamilton syringe and a 30-gauge needle using stereotactic apparatus (3 mm lateral and 3 mm posterior to the bregma). 5 weeks after implantation of tumor cells, the rats were subjected to MRI.

### Rat MR imaging

Rats (n = 5) with brain tumors were anesthetized with isoflurane (3% for induction, 1.5% maintenance) and a polyethylene catheter (PE50) was inserted into the tail vein for sucralose injection. Rats were transferred to a 9.4 T horizontal bore small animal MR scanner (Varian, Palo Alto, CA) and placed in a 35-mm diameter commercial quadrature proton volume head coil (m2m Imaging Corp., Cleveland, OH). Rats were kept under anesthesia (1.5% isoflurane in 1 l/min oxygen) and their body temperature maintained at 37 °C with the air generated and blowing through a heater (SA Instruments, Inc., Stony Brook, NY). Respiration and body temperature were continuously monitored using an MR compatible small animal monitoring system (SA Instruments, Inc., Stony Brook, NY).

Fast-spin-echo T2 weighted MRI was performed prior to the CEST experiments to determine the slice positioning of glioma. The parameters for T2 weighted imaging were: TR = 8000 ms, TE = 50 ms, FA = 90°, echo train length = 16, number of slices = 12, slice thickness = 2 mm, FOV = 30 × 30 mm^2^, matrix size = 128 × 128 and number of averages (NA) = 2. Following the whole brain T2 weighted brain imaging, a single slice 3 mm thick containing the tumorous region was acquired. This led to an in-plane resolution of 0.234 × 0.234 mm^2^. This same slice was used for all the subsequent sucCEST and Gd-DTPA experiments.

Chemical exchange saturation transfer imaging of rat brain tumor was performed using similar pulse sequence parameters as the phantom imaging experiments except FOV 30 × 30 mm^2^ B_1rms_ = 2.35 µT, saturation duration = 2 s and T1 delay = 8 s, slice thickness = 3 mm, NA = 4. After baseline imaging, the rats were injected with 2 ml of 200 mM sucralose solution at a rate of 0.2 ml/min through a catheter inserted in a tail vein (for 10 min). Following sucralose administration, CEST imaging was performed every 30 min.

### Gadolinium weighted imaging

Following the CEST imaging, a baseline T1-weighted image was acquired using the following parameters: FOV 30 × 30 mm^2^, TR = 6.22 ms, TE = 2.9 ms, FA = 20°, slice thickness = 3 mm, and NA = 12. Gd-DTPA (100 µl, 287 mg/ml) was injected as a bolus in 5 s through tail vein and another T1-weighted image was acquired to see Gd enhanced signal in the glioma.

### CEST image processing

First the acquired CEST weighted images were corrected for the B_0_ inhomogeneity and used to generate sucCEST [magnetization transfer ratio asymmetry, (*MTR*_asym_)] contrast map using Eq. [1].

1 $$MTR_{asym} \;(\% ) = 100 \times \left( {\frac{{S_{ - ve} - S_{ + ve} }}{{S_{0} }}} \right)$$

where *S*_−ve_, S_+ve_, and *S*_0_ are the B_0_ corrected MR signals at −1, 1 and 20 ppm, respectively. The CEST contrast map was further corrected for B_1_ inhomogeneity and overlayed onto anatomical proton image as false colors. Regions of interests (ROIs) were manually drawn on tumor and normal appearing brain regions. All image processing and data analysis were performed using software routines written in MATLAB (R2015b) as described in details elsewhere [16, 24].

## Results

### CEST effect from sucralose

**Fig. 1:**
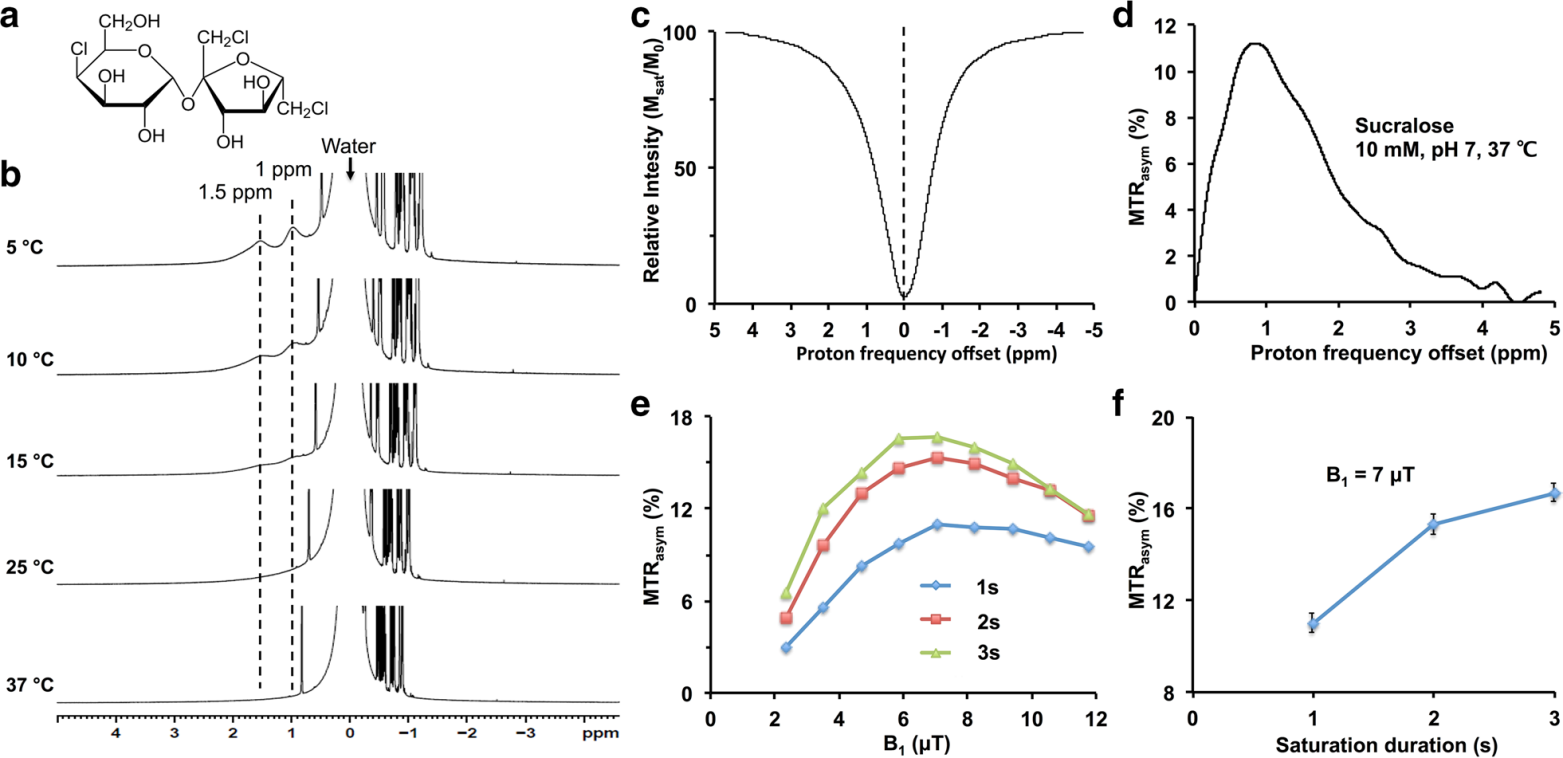
CEST effect from sucralose. **a** Chemical structure of sucralose shows the exchangeable –OH groups. **b** High resolution NMR spectrum of 200 mM sucralose solution in PBS shows two peaks from exchangeable hydroxyl protons (–OH) respectively at 1.0 and 1.5 ppm at 5 °C temperature. The peaks broadened with increase in temperature and were completely broadened at 37 °C. **c**, **d** Z-spectra and MTR_asym_ curves from 10 mM of sucralose (pH 7, 37 °C) show broad resonance (0–3 ppm), which peaked around 1 ppm. **e** The dependence of sucCEST contrast on saturation power and duration. The optimal B_1_ for the sucCEST contrast in the phantom was 7 µT. **f** The graph shows sucCEST contrast from 10 mM sucralose phantom at different saturation duration for a fixed B_1_ power (7 µT)

Sucralose, a chlorinated analog of sucrose, has five hydroxyl groups (–OH) that exchange with water protons in the solution (Fig. 1a). To determine the labile –OH proton resonance, high-resolution NMR spectra from a sucralose solution (200 mM, pH 7) were acquired at different temperatures (5, 15, 25, and 37 °C) on a 9.4 T NMR-spectrometer. At 5 °C, the spectrum showed two peaks at 1 and 1.5 ppm downfield to the water resonance in the NMR spectrum due to slower exchange of the –OH protons with water protons (Fig. 1b). With increase in temperature, both the hydroxyl peaks broadened due to the faster chemical exchange between the –OH protons and water protons until the –OH peaks became completely indistinguishable from baseline at 37 °C (Fig. 1b).

Z-spectra (Fig. 1c) and MTR_asym_ curves (Fig. 1d) from 10 mM sucralose solution (pH 7, 37 °C) showed a broad CEST effect between 0 and 3 ppm, which peaked at ~1 ppm downfield of water resonance. The sucCEST contrast (MTR_asym_ at 1 ppm) from 10 mM sucralose solution was measured at different pulse amplitudes (B_1_) and durations of the RF saturation pulse to determine the optimal saturation parameters. For all the saturation durations, the maximum sucCEST contrast was observed at a B_1_ of 7 µT in the solution (Fig. 1e). Generally, the sucCEST contrast increased with higher saturation duration for a given B_1_ and started to level off after 2 s (Fig. 1f).

### Concentration and pH dependence of sucCEST

**Fig. 2:**
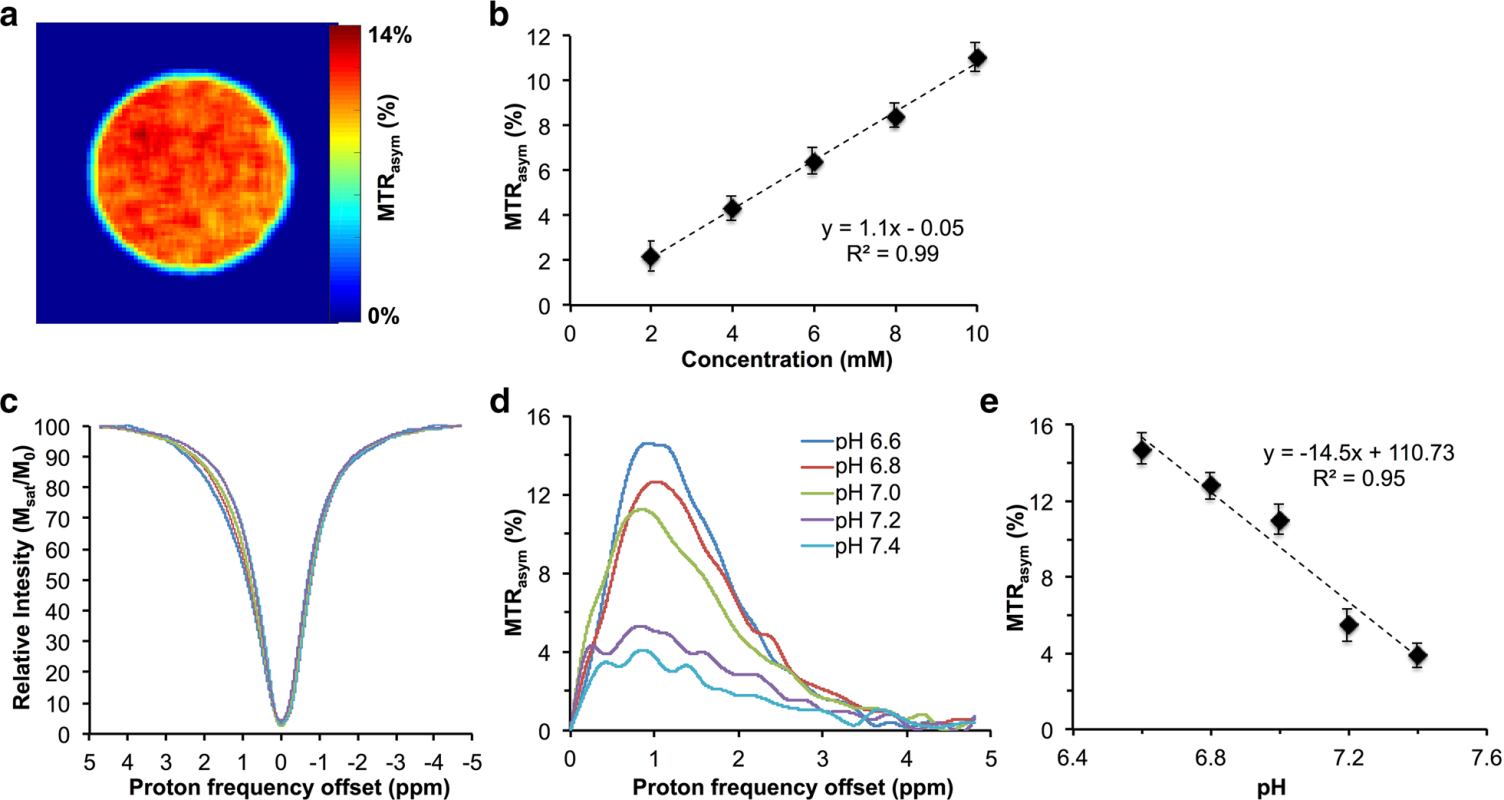
SucCEST map of sucralose. **a** sucCEST map obtained from 10 mM sucralose phantom at 37 °C shows ~11% contrast. **b** The graph shows linear increase in the sucCEST contrast with increase in sucralose concentration. **c**, **d** Z-Spectra and MTR_asym_ curves from different pHs show higher sucCEST contrast with decrease in the pH. **e** The graph shows a slope of 14.5% change in sucCEST per unit drop in the pH

The sucCEST map from 10 mM sucralose solution showed ~11% contrast at physiological temperature (37 °C) and pH (7) (Fig. 2a), and contrast was found to be linearly proportional to the sucralose concentration with a slope of 1.1% per mM of sucralose (Fig. 2b). Additionally, z-spectra and MTR_asym_ plots for 10 mM sucralose solution at different pHs (6.6, 6.8, 7.0, 7.2, 7.4), showed increased sucCEST contrast with decrease in pH (Fig. 2c, d). The sucCEST contrast increased 14.5% per unit decrease in the pH (Fig. 2e).

The phantom studies of Splenda showed a broad MTR_asym_ which peaked at ~1.5 ppm downfield of the water resonance. The Splenda CEST map generated at 1 ppm from 0.5% Splenda concentration showed ~20% contrast. The concentration-dependent graph showed a linear correlation of Splenda CEST contrast with Splenda concentration (Additional file 1: Figure S1).

### SucCEST experiments with normal rat

**Fig. 3:**
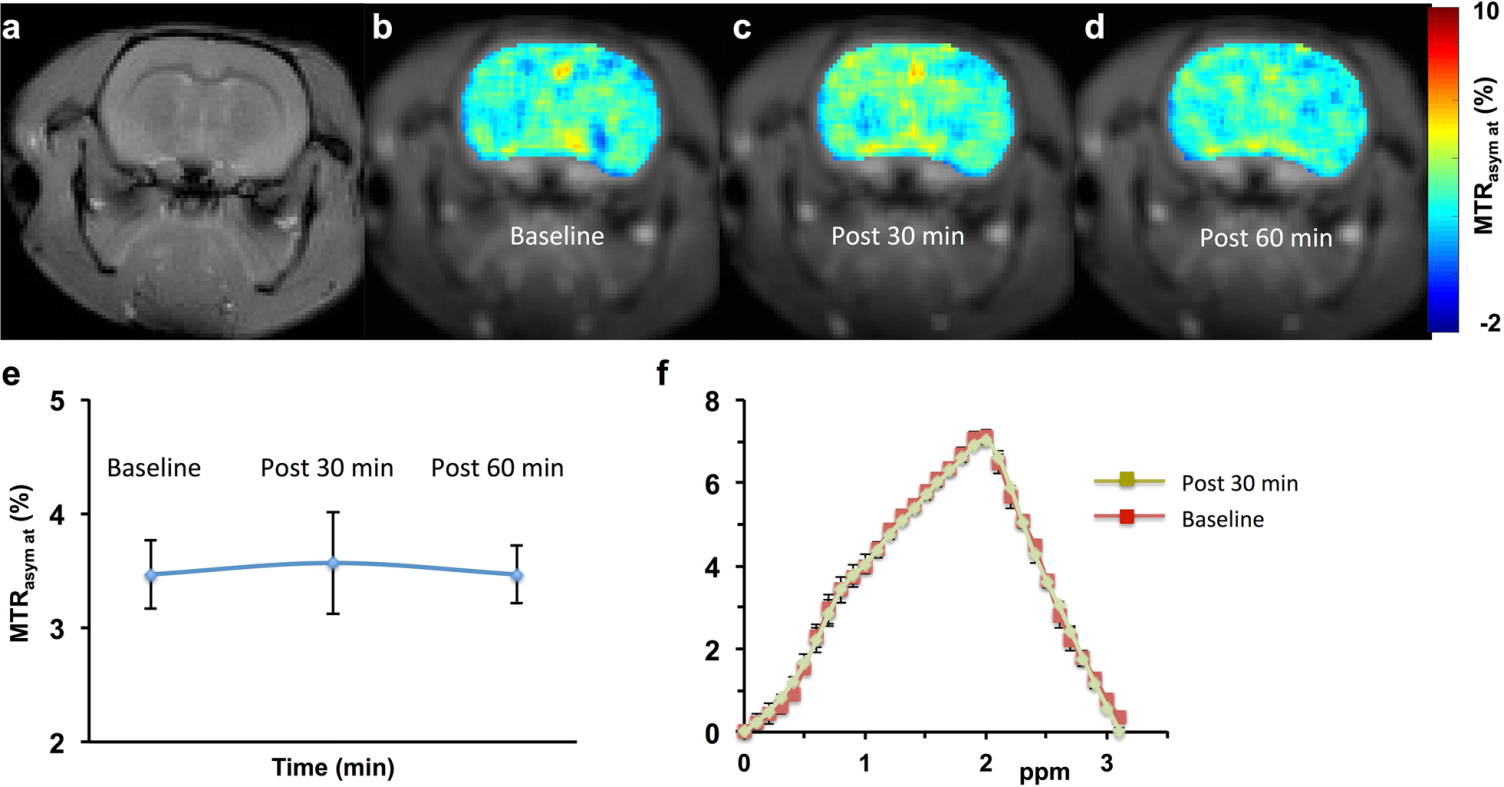
SucCEST imaging in normal rat brain. **a** T2 weighted image of the 3-mm thick coronal brain slice in a normal rat brain. **b** Baseline sucCEST contrast map using parameters (B1 2.35 µT, duration 2 s). **c**, **d** The images show no appreciable change in the sucCEST contrast following the administration of sucralose in normal rats. **e** Plot of MTR_asym_ (%) at 1 ppm from all the normal rats depicts no change in sucCEST in the normal brain. **f** Baseline and 30 min post sucralose asymmetry curves show no observable change in the MTR_asym_ curve at 1 ppm

In vivo study of sucCEST contrast from sucralose was evaluated in normal rats (n = 5) at 9.4 T horizontal bore MR scanner. CEST acquisitions with saturation frequency offsets from 0 to 3.6 ppm in step sizes of 0.2 ppm were used to generate z-spectra and MTR_asym_ maps from a coronal slice placed in the mid-brain region (Fig. 3a) with the following saturation pulse parameters: B1 = 2.35 µT, duration 2 s. In the baseline scans, ~3.5% contrast was observed at 1 ppm in the healthy rat brain (Fig. 3b). Following the baseline scans, sucralose (200 mM in PBS, pH 7) was injected intravenously (for 10 min, 0.2 ml/min) and CEST images from the rat brain were acquired at every 30-min time interval (Fig. 3c, d). SucCEST contrast was unchanged in the normal rat brain following sucralose administration suggesting that sucralose does not cross the intact BBB (Fig. 3e).

The MTR_asym_ curves over an ROI placed over the brain in the anatomical slice showed two peaks at 1 and 2 ppm downfield of water (Fig. 3f). The contrast at 1 ppm may be due to the endogenous –OH groups predominantly on glucose, myo-inositol or other metabolites containing hydroxyl protons [14, 17]. The 2 ppm peak in MTR_asym_ curve may be due to the –NH_2_ protons of creatine present in the brain [15, 37]. The MTR_asym_ curves obtained pre- and post- administration of sucralose did not change (Fig. 3f), demonstrating that normal BBB is impermeable to sucralose.

### SucCEST experiments with glioma rats

**Fig. 4:**
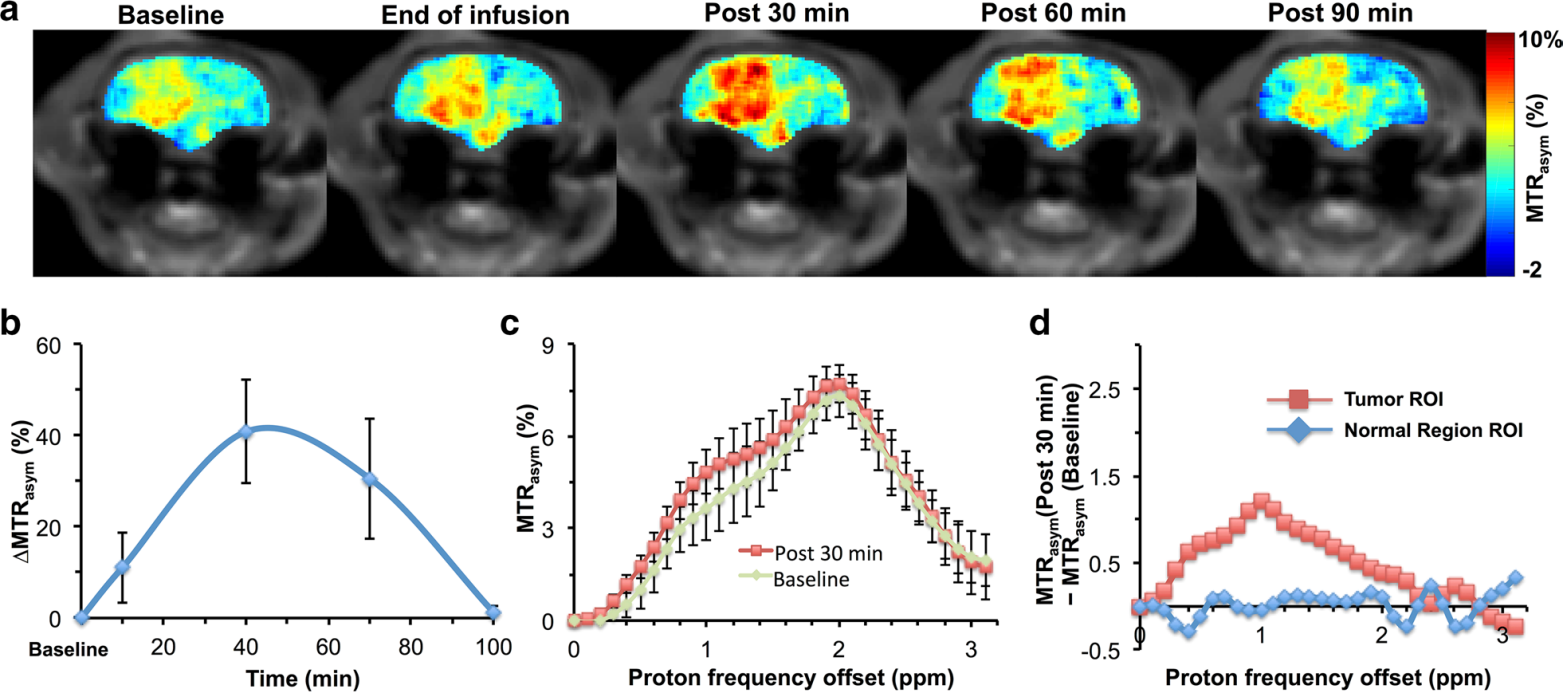
SucCEST map of a rat brain glioma. **a** sucCEST contrast map from a rat with glioma shows increased contrast in tumor region following intravenous injection of sucralose with the sucCEST contrast peaking at 30 min post injection. **b** The kinetics showing the average percentage change from the baseline in the sucCEST contrast from tumor rats at different time points, which peaks ~30 min post end of infusion. **c** MTR_asym_ curves generated from tumor ROI at baseline and post 30 min following the end of sucralose infusion show increased contrast at 1 ppm. **d** The normal brain, no signal change at 1 ppm post 30 min was observed in the MTR_asym_ curve

We evaluated the sucCEST contrast from sucralose in the 9L cell gliosarcoma rat brain tumor model (n = 5) in the same way as the normal rats. The anatomical MR slice was obtained in the tumor region before the CEST acquisitions (Fig. 4a). Immediately following the sucralose administration, the sucCEST contrast was found to be higher in the tumor region (Fig. 4b). The maximum increase in the sucCEST contrast was observed at 30 min post injection, (4.8%) (Fig. 4a). The elevated sucCEST contrast returned to the baseline value ~90 min post infusion (Fig. 4a–c). However, in the normal-appearing brain region of these rats, sucCEST contrast did not change appreciably over the course of 90 min, again indicating that sucralose did not get into the healthy regions of the brain (Fig. 4d).

The MTR_asym_ curves from tumor acquired pre- and post sucralose injection clearly observed a difference at 1 ppm (Fig. 4c), indicating that the change in the CEST contrast is due to the sucralose accumulation in the tumor region. Additionally, there was no significant difference between the asymmetry plots from the normal region of the brain pre- and post sucralose administration (Fig. 4d).

### Qualitative comparison of sucCEST images with Gd-enhanced images

**Fig. 5:**
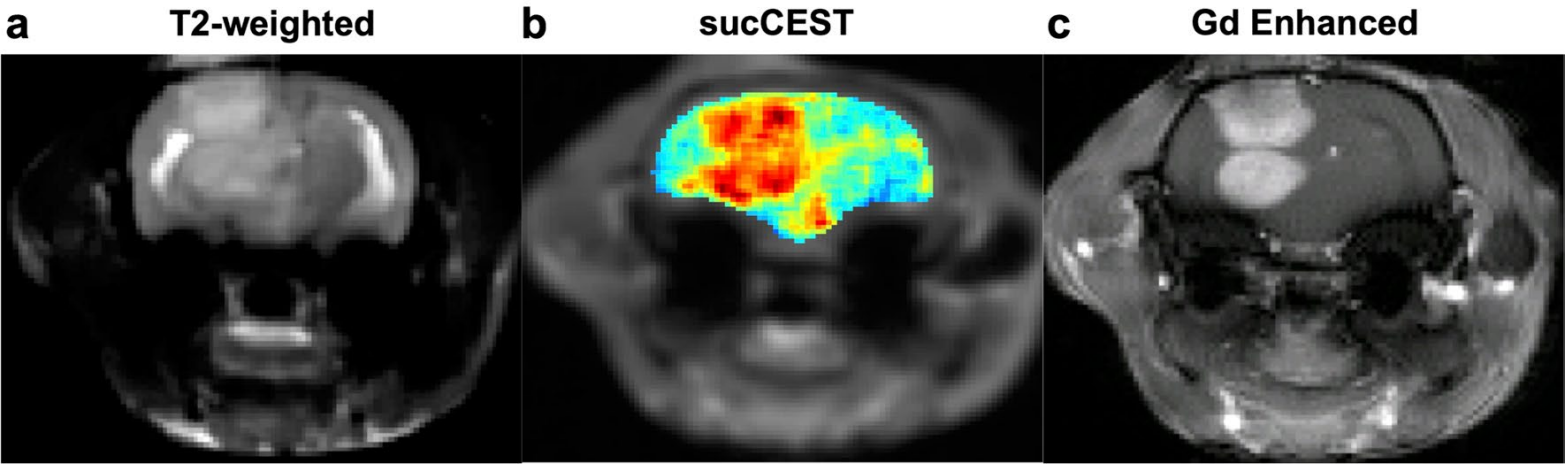
Gd-DTPA and sucCEST imaging. **a** T2 weighted image depicts the tumor as hyper-intense region. **b**, **c** The comparable gadolinium map is highlighting the tumor areas in the brain which is corroborating with the sucCEST map with sucralose injection

The sucCEST maps provided both visual and quantitative detection of tumor in rat brain and were qualitatively comparable with the Gd-DTPA enhanced images in the tumor region as shown in Fig. 5.

## Discussion

In the current study, we evaluated the use of the inexpensive, non-caloric sweetener sucralose as an MRI contrast agent based on chemical exchange to image tumors in vivo. In the normal rat brain, no change in the sucCEST contrast was observed following intravenous injection of sucralose, suggesting that sucralose does not cross the BBB and therefore can be used to image BBB disruption. Increased sucCEST contrast was observed in the tumor region, which is presumably due to the accumulation of sucralose in the extravascular extracellular space (EES) of the tumor. The brain tumor compromises the BBB and allows sucralose to enter the tumor EES. SucCEST sensitivity of 1.1% per mM sucralose translates to a ~1000-fold higher sensitivity than the direct detection with MRS, enabling the detection of millimolar concentrations.

Recently, d-glucose has been used as a CEST MRI contrast agent to image cancers (glucoCEST) [17, 29]. However, the interpretation of glucoCEST results might be intricate as the d-glucose is readily metabolized by both tumors and healthy tissue. Glucose analogues such as 2-deoxy-d-glucose (2-DG) and 2-fluoro-deoxy-d-glucose (FDG) were also shown to have higher CEST effect compared to glucose [31, 38]. This may be due to rapid conversion of glucose into lactate by the tumors [39] whereas 2-DG and FDG are not metabolized. As tumors are highly glycolytic, the injected glucose or pyruvate rapidly metabolize into lactate. Using the recently developed LATEST method to measure CEST contrast from lactate [18], it may be possible to map the glycolytic behavior of tumors as well as probe the kinetics of LDH activity in tumor. Another glucose derivative, 3-*O*-methyl-glucose (3-OMG) has recently been used as an MRI contrast agent to image cancer in orthotopic xenograft of a mammary adenocarcinoma model [40]. 3-OMG is taken up rapidly and preferentially by the tumor cells and stored. This contrasts with 2-DG and FDG, which undergo phosphorylation. Studies have shown that 3-OMG diffuses into normal brain tissue [30], though, limiting its use in the brain tumor imaging.

Unlike other CEST methods, sucCEST selectively highlighted the tumor. As sucralose is not metabolized in the body, the tumor sucCEST kinetics may be governed by the wash-in/wash-out of sucralose. Although we demonstrated the sucCEST in the brain tumor model, the method can potentially be useful to image other types of tumors and to monitor anti-tumor drug efficacy.

Sucralose phantom studies showed the highest CEST effect for saturation parameters of 7 µT and 3 s duration in vitro. However, in vivo T2 of water in the brain is much shorter (~40 ms) [41]. Short T2 leads to a large direct saturation effect when using relatively large B1 amplitudes for saturation and can obscure the desired CEST contrast [42]. Hence, we optimized the saturation B1 amplitude and duration separately in vivo and found that 2.35 µT and 2 s RF saturation pulse gave reliable 1 ppm MTR_asym_ in the brain.

The acute and sub chronic toxicity effect of oral administration of sucralose has been evaluated previously in animals, and no sucralose-related adverse effects were observed following the dietary administration of sucralose in mice (16 g/kg), rats (10 g/kg) and dogs (900 mg/kg/day) [43]. Another study in human volunteers showed no adverse effect of acute or chronic oral dosage of sucralose [44]. These studies established that sucralose is non-toxic following high acute oral administration. While other studies reported no toxic effect of intravenous administration of sucralose at lower dosage (20 mg/kg) in mice and rats [45, 46], we are not aware of any published toxicity result at the intravenous administration dosage (500 mg/kg) used in the present study. In this preliminary study, we did not observe any adverse effect of intravenously injected sucralose in normal or tumor-bearing rats. However, immediately following the start of the infusion of sucralose, the respiration rate of the rats was found to increase by ~20 breath/min before getting back to the baseline rate in ~2 to 3 min. For the future studies, the optimum concentration and the rate of injected sucralose may be explored.

We suggest that sucCEST with intravenous infusion of sucralose can serve as a diagnostic and therapeutic response monitoring tool in preclinical studies of tumors. While it is possible to use this method to study cancer patients at ultrahigh fields, more detailed toxicity studies of intravenously administered sucralose are required before undertaking such studies.

## Conclusion

This inexpensive, non-caloric sweetener can be readily used for routine examination of various tumors on ultra-high field MRI scanners based on contrast generated from the chemical exchange of its labile hydroxyl protons with water. In addition, it can potentially be used to study BBB derangements, noninvasively. This preliminary study paves the way for the development of sucralose and other sucrose derivatives as MRI contrast agents for a variety of human clinical imaging applications as well as to monitor therapeutic response.

### Additional file

**Additional file 1: Figure S1.** CEST imaging of Splenda. **a**, **b** Z-spectra and MTR_asym_ curves show a broad asymmetry, which peaks at ~1.5 ppm. **c** The CEST map from 0.5% Splenda shows ~20% contrast at 1 ppm. **d** SplendaCEST contrast was linearly proportional to the Splenda concentration.

## Abbreviations

CEST: chemical exchange saturation transfer
PET: positron emission tomography
^18^FDG: ^18^fluoro-2-deoxy-glucose
MRI: magnetic resonance imaging
GBCA: gadolinium based contrast agents
MRS: MR spectroscopy
Gd_DTPA: gadolinium-diethylenetriamine pentaacetic acid
PBS: phosphate buffered saline
BBB: blood–brain barrier
OH: hydroxyl groups
MTR_asym_: magnetization transfer ratio asymmetry
FOV: field of view
TR: repetition time
TE: echo time
FA: flip angle
NA: number of averages
ROIs: regions of interests
MTR_asym_: magnetization transfer ratio asymmetry
EES: extravascular extracellular space
2-DG: 2-deoxy-d-glucose
FDG: 2-fluoro-deoxy-d-glucose
3-OMG: 3-*O*-methyl-glucose
