# Supplementary figures and images for: Non-caloric sweetener provides magnetic resonance imaging contrast for cancer detection
Source: J Transl Med. 2017 May 30;15:119. doi: 10.1186/s12967-017-1221-9 (PMC5450413; doi:10.1186/s12967-017-1221-9)

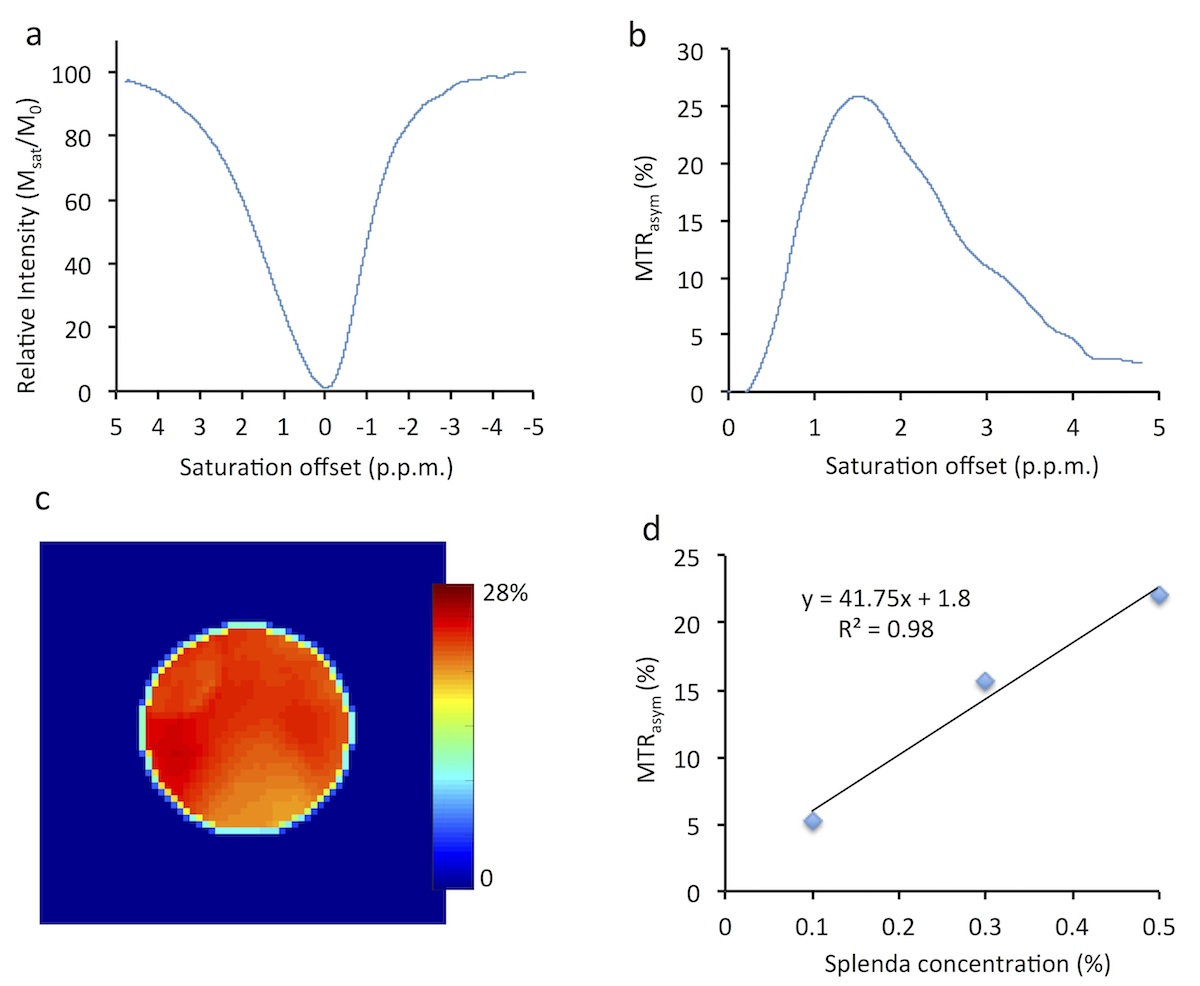

Supplement: Supplementary file 1 — Additional file 1: Figure S1. CEST imaging of Splenda. a, b Z-spectra and MTRasym curves show a broad asymmetry, which peaks at ~1.5 ppm. c The CEST map from 0.5% Splenda shows ~20% contrast at 1 ppm. d SplendaCEST contrast was linearly proportional to the Splenda concentration. [file 12967_2017_1221_MOESM1_ESM.jpg]
